# Arylsulfatase A pseudodeficiency in Mexico: Enzymatic activity and haplotype analysis

**DOI:** 10.1002/mgg3.1305

**Published:** 2020-05-19

**Authors:** Jesús A. Juárez‐Osuna, Sandra C. Mendoza‐Ruvalcaba, Angela Porras‐Dorantes, Thiago D. Da Silva‐José, José E. García‐Ortiz

**Affiliations:** ^1^ Doctorado en Genética Humana Centro Universitario Ciencias de la Salud Universidad de Guadalajara Guadalajara México; ^2^ Laboratorio de Diagnóstico Bioquímico de Enfermedades Lisosomales División de Genética Centro de Investigación Biomédica de Occidente (CIBO) Instituto Mexicano del Seguro Social (IMSS) Guadalajara México; ^3^ Dirección de Educación e Investigación en Salud UMAE Hospital Gineco‐obstetricia CMNO‐IMSS Guadalajara México

**Keywords:** Arylsulfatase A, enzyme activity, haplotypes, metachromatic leukodystrophy, pseudodeficiency

## Abstract

**Background:**

Metachromatic Leukodystrophy (MLD, OMIM 250100) is a neurodegenerative disease caused by mutations in the *ARSA* gene (OMIM 607574) that lead to deficiency in Arylsulfatase A (ASA). ASA pseudodeficiency (PD‐ASA) is a biochemical condition that substantially diminishes ASA activity but is not associated with clinical manifestations. PD‐ASA is associated with the c.1055A>G (p.Asn352Ser) (rs2071421) and c.*96A>G (rs6151429) variants, which have an estimated frequency of 2% in the population.

**Objective:**

To determine the activity of Arylsulfatase A and to identify variants and haplotypes in the *ARSA* gene in Mexican individuals with pseudodeficiency.

**Methods:**

Two‐hundred apparently healthy individuals were included to determine the enzymatic activity of ASA in leukocytes by spectrophotometric analysis, and identification of the PD‐ASA alleles was performed by PCR‐RFLP assays. Genotypes were confirmed by semi‐automated Sanger sequencing. Haplotypes were constructed using Arlequin v.10.04, and linkage disequilibrium analysis was performed with Cube X.

**Results:**

The enzymatic activity of ASA was determined to be 1.74–2.09 nmol/mg protein/min and later correlated with genotypes and haplotypes. For the (p.Asn352Ser) variant, we found 126 (0.63) individuals with the AA genotype, 62 with AG (0.31) and 12 with GG (0.06); the frequency of the polymorphic allele was 0.215 (86 alleles, 21.5%), and the variant was in HWE (*p* = .2484). The variant c.*96A>G was also in HWE (*p* = .2105): 185 individuals (0.925) with the AA genotype, 14 (0.07) with AG, and 1 (0.005) with (GG), with a frequency of 0.04 (4%) for the polymorphic allele. The inference of haplotypes resulted in 312 (0.78) AA, 72 (0.18) GA, and 16 (0.04) GG haplotypes. The AG haplotype was not found. The variants were found to be in linkage disequilibrium (D' = 1). Of the nine possible diplotypes, AA/AG, AA/GG, and AG/GG were not found, in concordance with the hypothesis that the G allele of c.*96A>G does not occur in the absence of the G allele of c.1055A>G. We found a slight correlation between ASA biochemical activity and variants, mainly due to the G allele of c.*96A>G in either genotypes or haplotypes.

**Conclusions:**

In Northwestern Mexico, the presence of PD‐ASA alleles was biochemically and molecularly determined, and the frequencies were found to be in HWE. The frequency of PD‐ASA for the North Western Mexican mestizo is 8%.

## Graphical abstract

Determine the activity of arylsulfatase A and identify variants in the ARSA gene in Mexican individuals with pseudodeficiency of arylsulfatase A.

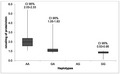

## 1 INTRODUCTION

Metachromatic leukodystrophy (MLD) (OMIM: 250,100) is an autosomal recessive, chronic, hereditary, neurodegenerative disease with a general prevalence of 1 per 60,000 to 1 per 70,000 individuals (Heim et al., 1997; Poorthuis et al., 1999). It is caused by deficiency in the enzyme Arylsulfatase A (ASA; E.C. 3.1.6.8) due to genetic variants in the *ARSA* gene (Cesani et al., 2016) that lead to the accumulation of cerebroside sulfate, a glycolipid that forms part of myelin membranes (Gieselmann, Polten, Kreysing, & von Figura, 1989). Interestingly, 1 of every 50 healthy individuals in the general population (worldwide) has a substantial ASA deficiency without clinical signs of the disease; these individuals’ residual activity is reduced to approximately one tenth of the normal enzyme activity (Shen, Li, Waye, Francis, & Chang, 1993), indicating that this low enzymatic activity is sufficient to maintain a normal metabolism, with no accumulation of sulfates and no excretion of them in urine (Gieselmann, 2007).The term pseudodeficiency is misleading; however, although the deficiency is real, the classic MLD phenotype is not observed (Penzien et al., 1993)**.** The ASA pseudodeficiency (ASA‐PD) has been associated mainly with the presence of two variants in *cis*. The first variant, c.1055A>G (p.Asn352Ser) (rs2071421), is a transition from adenine to guanine, responsible for the substitution of an asparagine for serine at amino acid 352 (exon 6), causing the loss of one of the three N‐glycosylation sites in ASA; therefore, the produced enzyme has only two oligosaccharide side chains, whereas the normal enzyme has three. The second variant, c.*96A>G (rs6151429), leads to loss of the polyadenylation signal in exon 8, which is required to complete the synthesis of a 2.1 kb mRNA (Gieselmann et al., 1989). The alleles have been previously reported to be in linkage disequilibrium and, despite the difference in their frequencies, there is a hypothesis that the G allele of c.*96A>G will always be present with the G allele of c.1055A>G (Ben Halim et al., 2016). There are currently no population studies in Mexico that describe the frequencies of the variants mentioned in the literature, however, there are reports that highlight the importance of these alleles due to their high frequencies in the general population.

## 2 MATERIALS AND METHODS

**FIGURE 1:**
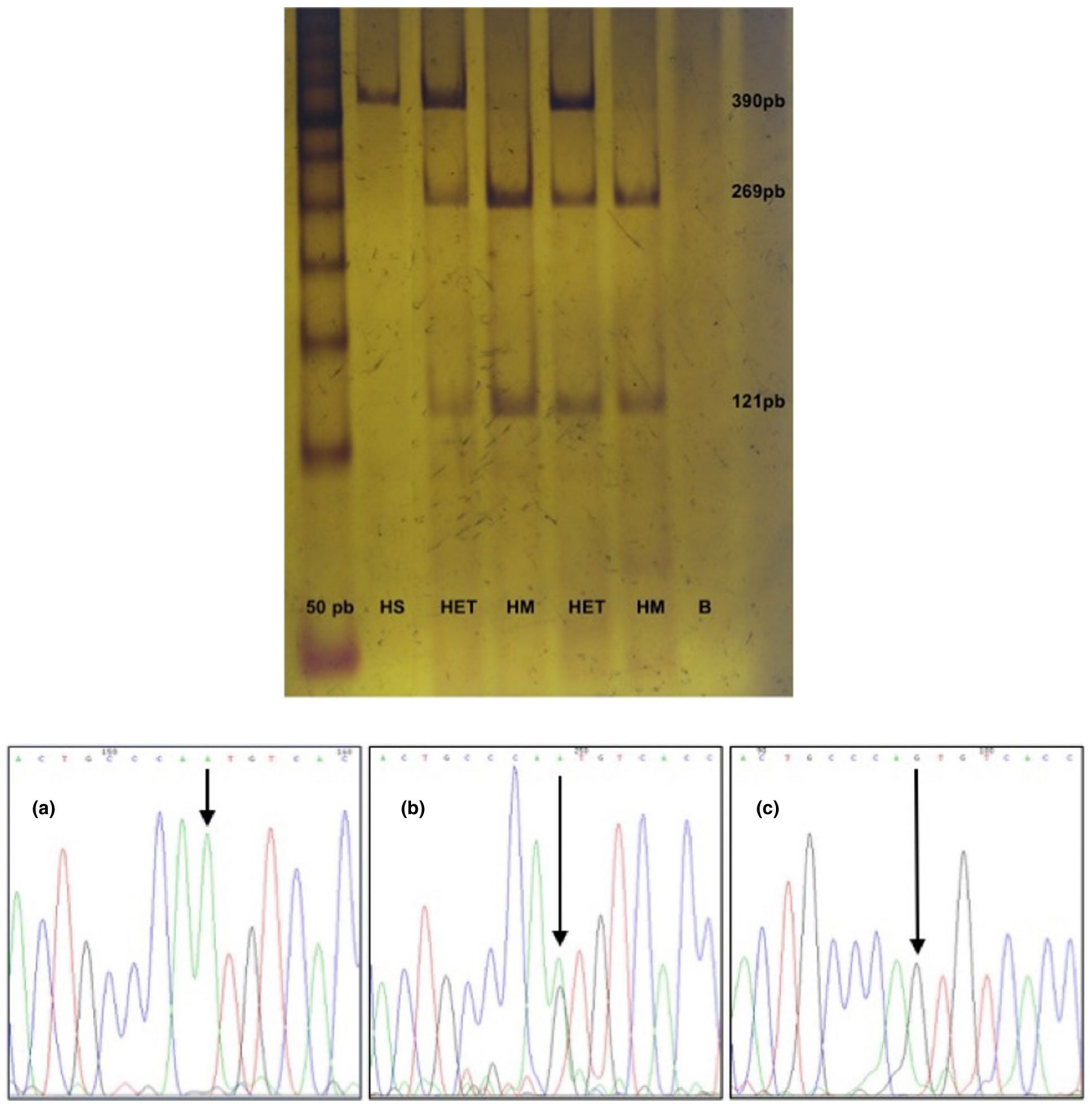
Molecular analysis of variant c.1055A>G (p.Asn352Ser) (rs2071421): (A) 6% polyacrylamide electrophoresis, which shows the restriction products with BsrI for the identification of variant c.1055A>G. The first lane corresponds to the molecular weight marker (mpm) that this time was 50 bp, the second lane (HS) is a wild homozygous (390 bp), the third and fifth lane (HET) are samples of heterozygous (390 bp, 269pb, 121pb), while lanes 4 and 6 (HM) are homozygous for variant and lane 7 (B) corresponds to blank. (B) Electropherograms representing genotypes for variant c.1055A>G. (a) Corresponds to the wild homozygous genotype (AA), (b) heterozygous genotype (AG), and (c) variant homozygous genotype (GG)

**FIGURE 2:**
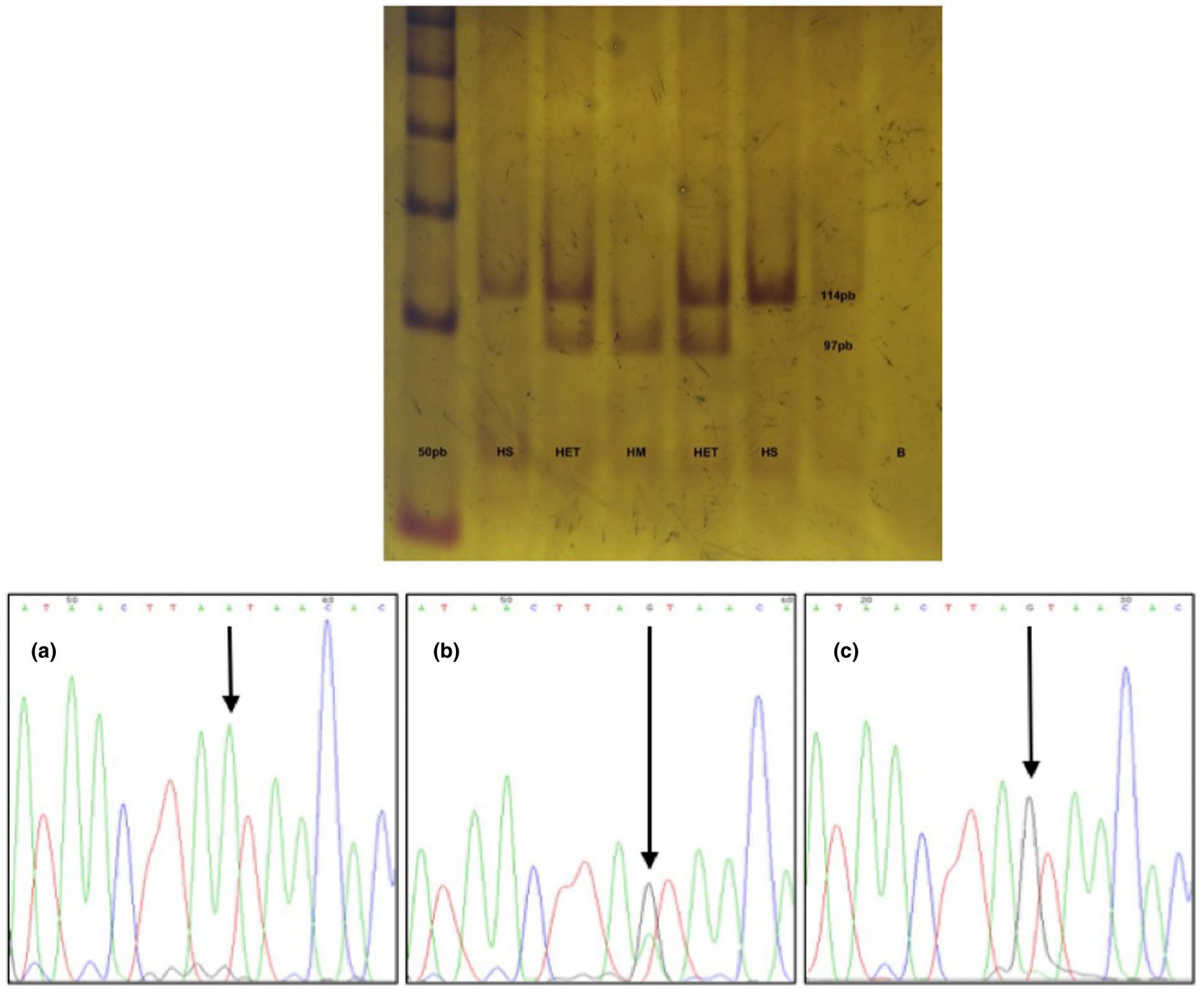
Molecular analysis of variant c.*96A>G (rs6151429): (A) 6% polyacrylamide electrophoresis, which shows the restriction products with DdeI for the identification of variant c.*96A>G. The first lane corresponds to the molecular weight ladder that this time was 50 bp, the second and sixth lane (HS) is a wild homozygote (114 bp), the third and fifth lane (HET) are samples of heterozygous (114, 97 bp, and in theory a 17 bp fragment that is lost during electrophoretic running), while lane 4 (HM) is homozygous for variant and lane 8 (B) corresponds to blank. (B) Electropherograms representing genotypes for variant c.*96A>G. (a) Corresponds to the wild homozygous genotype (AA), (b) heterozygous genotype (AG), and (c) variant homozygous genotype (GG)

After informed consent was obtained, peripheral blood was collected from 200 Northwestern Mexican mestizo individuals (112 men, 88 women) using a vacuum tube with EDTA. All the individuals were between 18 and 34 years old at the time the sample was taken and were students of the Centro Médico Nacional de Occidente (CMNO), all of them apparently healthy (asymptomatic), Mexican ancestry was considered when all parents and grandparents were born in Mexico. Genomic DNA was extracted by the salting‐out technique (Miller, Dykes, & Polesky, 1988). Leukocytes were obtained by precipitation and lysis of erythrocytes with citric acid, sodium citrate, and D‐glucose (ACD) (Stanbury, Wyngaarden, & Fredrickson, 1972), and the total protein concentration was determined with the Lowry method (Lowry, Rosebrough, Farr, & Randall, 1951). The determination of enzymatic activity in ASA leukocytes was performed by a modification in the Percy assay (Percy & Brady, 1968) with 4‐nitrocatechol sulfate dipotassium salt as the substrate (Sigma^®^ LifeScience; CAS:14528‐64‐4). Confidence intervals were calculated with the formula: x̅ ± Za/2 * σ/√(n). Analysis of the variants was performed by PCR‐RFLP assay. For the c.1055A>G (p.Asn352Ser) (rs2071421) variant, a 390 bp fragment was amplified by PCR with the modified primers F‐5' C**C**/GCAACCTTGATGGCGAGT 3' and R‐5' AAGCACTGCACATACCTG**C**/GG 3' and the enzyme *Bsr*I (catalog # R0527S; Time‐Saver^™^, BioLabs Inc.) was used for digestion (Figure 1a). In the case of the c.*96A>G (rs6151429) variant, a modified primer was used. A 114 bp fragment was amplified by PCR with the primers F‐5'GGTTTGTGCCTGATAACGTAA 3' and R‐5' TTCCTCATTCGTACCACAGG 3'. For digestion, the enzyme *Dde*I (catalog # R0175S; Time‐Saver^™^, BioLabs^®^ Inc.) was used (Figure 2a) (Barth, Ward, Harris, Saad, & Fensom, 1994). Genotypes were confirmed by Sanger sequencing (Big Dye Terminator v3.1 Cycle Sequencing Kit; Applied Biosystems) with an ABI Prism 310 Sequencer (Applied Biosystems) (Figures 1b and 2b), GeneBank reference sequence NG_009260.2 was considered. Finally, haplotypes were constructed using Arlequin v.3.5, and linkage disequilibrium analysis was performed with Cube X.

## 3 RESULTS

### 3.1 Enzymatic activity

The activity values of the 200 subjects were distributed nonparametrically (Shapiro‐Wilk test). Taking into account the enzymatic activity, 16 individuals with pseudodeficiency were identified. Within the descriptive statistics, an average of 1.91 nmol/mg protein/min was obtained, with a standard deviation of 0.85 nmol/mg protein/min and a 95% confidence interval of 1.74–2.09 nmol/mg protein/min. A pseudodeficient individual with GG/GG diplotype (GG genotype for both variants) had an enzymatic activity of 0.11 nmol/mg protein/min, corresponding to 13% relative to normal values. This enzymatic activity could represent a positive result in a biochemical diagnosis.

### 3.2 Genotyping

For the c.1055A>G (p.Asn352Ser) (rs2071421) variant, we found 126 (0.63) individuals with the AA genotype, 62 with AG (0.31), and 12 with GG (0.06). The frequency of the polymorphic allele was 0.215 (86 alleles, 21.5%), and the variant was found to be in HWE (*p* = .2484). The variant c.*96A>G (rs6151429) was also in HWE (*p* = .2105); in this case, 185 individuals (0.925) had the genotype AA, 14 (0.07) had AG, and 1 (0.005) had GG, with a frequency of 0.04 (4%) for the polymorphic allele. The inference of haplotypes resulted in 312 (0.78) AA, 72 (0.18) GA, and 16 (0.04) GG haplotypes. The AG haplotype was not found (Table 1). The variants were found to be in linkage disequilibrium (D' = 1).

**TABLE 1:** Genotype, allele, and haplotype frequencies of ASA‐PD alleles in 200 healthy Mexican mestizo individuals

*n* = 200	Genotype frequencies		Allele frequencies		Haplotype frequencies	
c.1055A>G *p* = .2484 (HWE)	A/A	126 (0.63)	A	314 (0.785)	A A	312 (0.78)
	A/G	62 (0.31)	G	86 (0.215)	A G	0 (0.0)
	G/G	12 (0.06)			G A	72 (0.18)
c.*96A>G *p* = .2105 (HWE)	A/A	185 (0.925)	A	384 (0.96)	G G	16 (0.04)
	A/G	14 (0.07)	G	16 (0.04)		
	G/G	1 (0.005)				

### 3.3 Variants versus enzymatic activity

**FIGURE 3:**
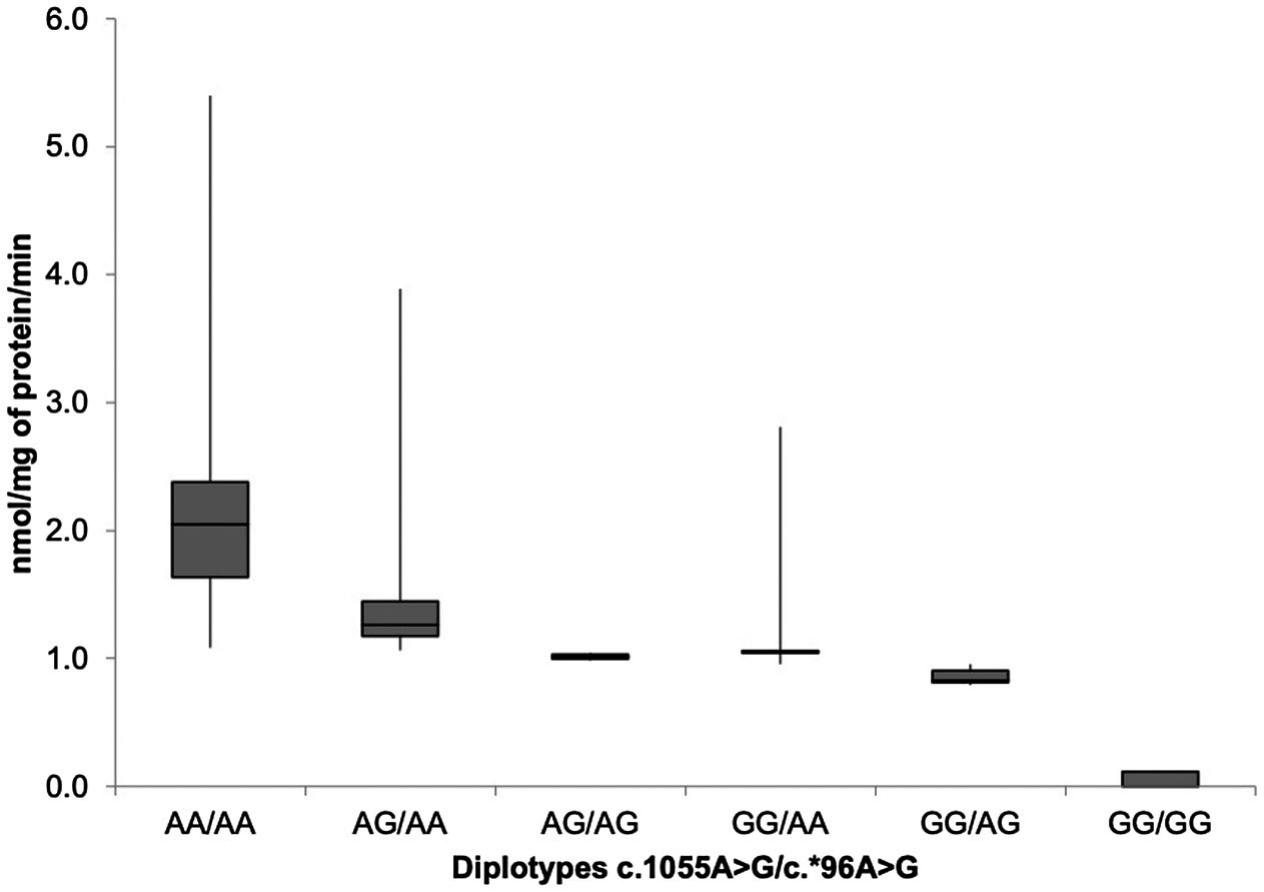
Residual enzymatic activity of Arylsulfatase A by diplotypes

**FIGURE 4:**
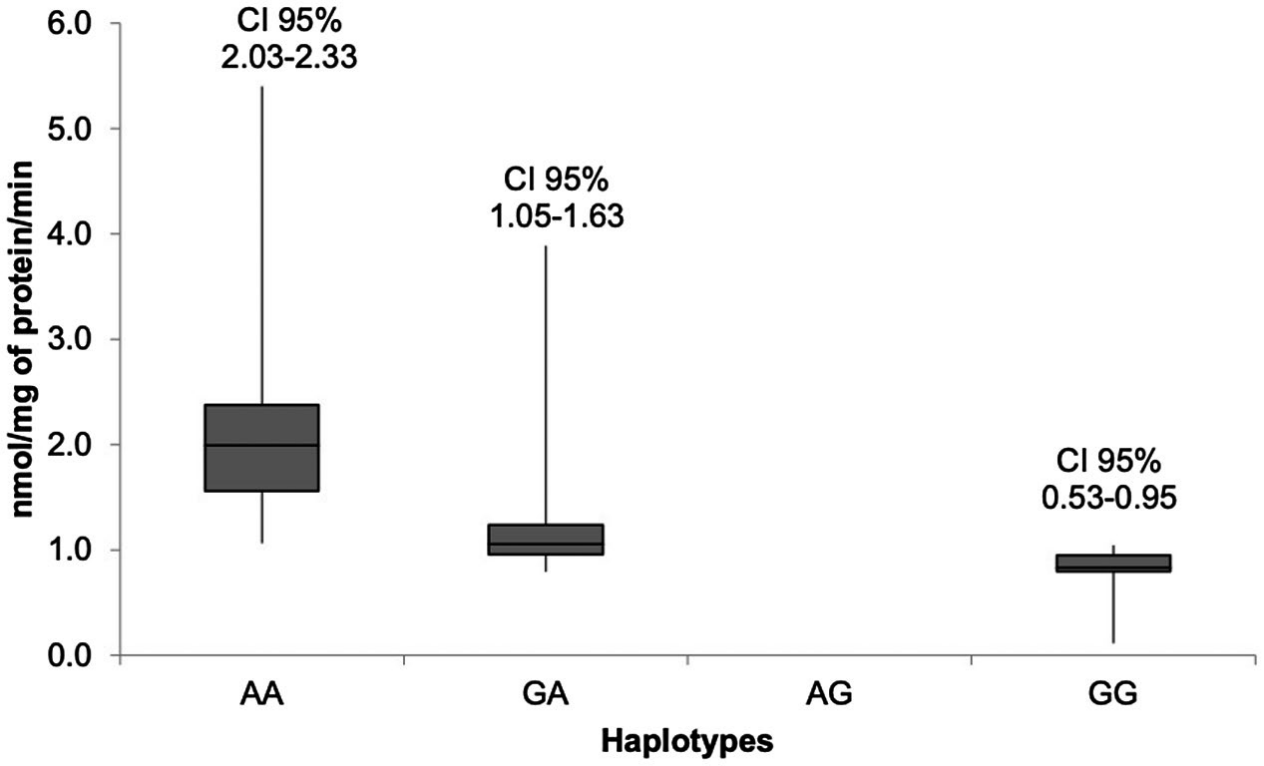
Residual enzymatic activity of Arylsulfatase A by haplotypes

Revealing the impact of the presence of ASA‐PD variants on the enzymatic activity of ASA, the graphs (Figure 3) show the combinations of genotypes or diplotypes (c.1055A>G/c.*96A>G) depending on the ASA activity. Of the nine possible diplotypes, AA/AG, AA/GG, and AG/GG were not found. It was expected that the G allele of c.*96A>G does not occur in the absence of the G allele of c.1055A>G. The graph shows a slight trend of ASA activity according to the present variant. It is important to note that the G allele of c.*96A>G indicates that the ASA activity decreases considerably. In the same way as with the diplotypes, in the graph (Figure 4), the inferred haplotypes are represented against the ASA activity, and the haplotype of interest (GG) presents less activity than the other haplotypes, whereas the haplotype AG was not found.

## 4 DISCUSSION

In our population, a pseudodeficient individual had an activity corresponding to 13% (0.11 nmol/mg protein/min) enzymatic activity relative to the lower limit preset as a control. As shown in the literature, the values of pseudodeficiency can reach up to 10% of enzymatic activity without presenting any clinical symptoms (Shen et al., 1993). Variant c.*96A>G implies the loss of a polyadenylation signal, so the average life of the mRNA is shorter, however, the low amount of protein produced is viable and prevents the accumulation of substrate and therefore the appearance of symptoms. Here, we show the correlation between genotypes and enzymatic activity and adds some light in the intriguing impact of polymorphic variants on enzymatic activity. Although no molecular assay was performed to determine if the alleles were present in cis, this inference was taken into account given that previous references mention that the G allele of c.*96A>G allele is always present with a G allele of c.1055A>G. In this study, 16 of 200 individuals showed pseudodeficiency, we can say that there is a frequency of 8% of ASA‐PD in the Northwestern Mexican mestizo population. This estimation is important given the complications that might occur in diagnosis of Metacromatic Leukodystrophy. In Spain, a frequency of 12.7% was reported, with 3.6% homozygous (Chabas et al., 1993); in Brazil, a frequency of 7.9% was established (Pedron, Gaspar, Giugliani, & Pereira, 1999), and the highest frequency was found in Australia, with 20%. The study of this condition improves the diagnostic accuracy and allows us to rule out cases with clinical suspicion of MLD reducing the time to the definitive diagnosis. The frequency of 8% may seem slightly high, however, the frequencies we observed are similar to those previously reported.

## 5 CONCLUSION

The frequency of the A allele for (p.Asn352Ser) (rs2071421) was 0.785, and 0.215 for the G allele, whereas for the c.*96A>G (rs6151429) variant, the A allele frequency was 0.94, and 0.04 for the G allele. The presence of the G allele of the c.*96A>G variant causes the enzyme activity to be reduced. We found in a healthy general population an ASA enzymatic activity of 13% and the GG/GG diplotype, suggesting that there are enzymatic values close to 10% due to pseudodeficiency alleles. The identification of pseudodeficient alleles helped us to provide accurate diagnosis in a short period of time.

## CONFLICT OF INTEREST

The authors listed declare no conflict of interest.

## AUTHORS CONTRIBUTIONS

Conceptualization, Methodology: Jesús A. Juárez‐Osuna & José Elías García‐Ortiz; Sample collection: Jesús A. Juárez‐Osuna, Angela Porras‐Dorantes, Thiago Donizete Da Silva‐José; Logistics, Equipment, Experiment design, Experiments, and Sanger Data Analysis for all individuals here assessed: Jesús A. Juárez‐Osuna, Sandra C. Mendoza‐Ruvalcaba; Data curation, Data analysis, Investigation: Jesús A. Juárez‐Osuna & José Elías García‐Ortiz; Project administration, Resources, Software: Jesús A. Juárez‐Osuna & José Elías García‐Ortiz; Writing‐original draft, Writing‐review, & editing: Jesús A. Juárez‐Osuna & José Elías García‐Ortiz; All authors read and approved the final manuscript.

## Data Availability

All data included in this study are available upon request by contact with the corresponding author.
